# Two-generation exposure to a high-fat diet induces the change of salty taste preference in rats

**DOI:** 10.1038/s41598-023-31662-0

**Published:** 2023-04-07

**Authors:** Saranya Serirukchutarungsee, Ippei Watari, Masataka Narukawa, Katarzyna Anna Podyma-Inoue, Pornchanok Sangsuriyothai, Takashi Ono

**Affiliations:** 1 Department of Orthodontic Science, Graduate School of Medical and Dental Sciences, Tokyo Medical and Dental University (TMDU), Yushima 1-5-45, Bunkyo City, Tokyo, 113-8510 Japan; 2 Department of Pedodontics and Preventive Dentistry, Faculty of Dentistry, Srinakharinwirot University, Bangkok, Thailand; 3 Department of Food and Nutrition, Kyoto Women’s University, Kyoto, Japan; 4 Department of Orthodontics, Faculty of Dentistry, Chulalongkorn University, Bangkok, Thailand; 5 Department of Biochemistry, Graduate School of Medical and Dental Sciences, Tokyo Medical and Dental University (TMDU), Tokyo, Japan

**Keywords:** Developmental biology, Nutrition disorders, Physiology, Metabolism

## Abstract

High-fat diet (HFD) leads to multiple complications, including taste alteration. This study observed the effect of a two-generation exposure to an HFD on the peripheral taste system in offspring. Ten pregnant Wistar rats were assigned a standard diet (SD) (n = 5) or HFD (n = 5) from day 7 of pregnancy through the lactation. Thirty-six male and female 3-week-old offspring were measured for body weight and blood glucose level, and the circumvallate papillae were collected. The other twenty-four 3-week-old offspring were weaned on the same diet as their mothers and raised individually. The taste preference behaviors were studied using the two-bottle taste preference test and analyzed five basic tastes (sweet, bitter, umami, sour, and salty). The expressions of epithelial sodium channel alpha subunit (ENaCα) and angiotensin II receptor type 1 (AT1) in the circumvallate papilla were analyzed by immunohistochemical staining and reverse transcription-quantitative polymerase chain reaction (RT-qPCR). We found increased body weight and salty taste preference of offspring from the HFD group in both sexes. Correspondingly, the AT1 level of the taste bud cells significantly increased in 3-week-old female offspring from the HFD group. An increase in AT1 levels may be a risk factor for changes in salty taste preference.

## Introduction

Imbalanced nutrition during pregnancy and lactation severely affects long-term irreversible outcomes in offspring owing to interference during a critical period of fetal growth^1^. Fetal gene expression was influenced by genetics and epigenetics and results in the development of adverse effects in the offspring in later life^1,2^. Accordingly, the Developmental Origin of Health and Disease (DOHaD) concept was introduced and considered as the underlying mechanism of the influence of environmental factors during early development on the risk of non-communicable diseases in later life^3–6^. For instance, birth weight is associated with central body fat distribution, metabolic syndrome, ischemic cardiovascular disease, insulin resistance, and type 2 diabetes^7^.

A high-fat diet (HFD) has been reported to induce metabolic disorders in rodents that simulate a similar effect in humans^8,9^. Rodents fed an HFD developed insulin resistance, alteration in translocation of the muscular glucose transporter-4, and impairment of glucose uptake in muscle^8,10^. Moreover, adipocyte hypertrophy, hyperplasia, and hepatic steatosis were observed. A higher number and size of adipocytes eventually caused increased inflammatory genes and plasma leptin levels^8^. Interestingly, the influence of an HFD on the metabolic system can occur after a few weeks of HFD consumption^8,9^.

Maternal HFD exposure during pregnancy and lactation induces an irreversible effect in offspring^11,12^. Pups from healthy mother rats exposed to the HFD during late pregnancy and lactation displayed the alteration in metabolic hormones^11^. This result suggests that the effect of an HFD could be transferred to the offspring. Interestingly, post-weaning HFD exacerbated the adverse effect of maternal HFD^13–15^. It is important to comprehend that not only fetal life but also postnatal exposure to an HFD increases the risk of complications. Understanding the underlying pathologies of life and acknowledging the possibility of preventions will provide a significant advantage in reducing the burden of various metabolic non-communicable diseases in adults^7^. Moreover, it can protect against transgenerational complication cycles across multiple generations.

Food consumption patterns explicitly regulate energy intake and influence obesity^16,17^. Previous studies have indicated that eating behavior and weight gain are the results of the interaction between genetic and external environments^18^. Complex factors, such as sensory factors, cognitive factors, brain mechanisms, and satiety/hunger signals, influence eating behavior^19^. Taste, a sensory system, plays a major role in food intake behavior^17,20,21^. The pleasant taste of food could promote satiety signals and thus, was interpreted as the hedonic or rewarding value of food, which results in increasing appetite^19^. Therefore, abnormal taste perception possibly contributes to excessive eating and results in increased body fat accumulation.

Several studies have reported that imbalanced nutrition affects an offspring’s eating patterns and leads to taste alterations^22–25^. Offspring from rats exposed to the palatable food high in energy, fat, sugar, and salt during gestation and lactation developed hyperphagia and preferred foods high in fat, sugar, and salt more than offspring from chow diet-fed rats^24^. In addition, previous studies have observed a higher sweet taste preference in offspring of HFD-fed mother rats than in offspring of control mother rats^22,23,25^. It is essential, therefore, to consider that taste alteration in offspring could be a critical factor for excessive energy intake. To our knowledge, no previous studies have investigated the effect of two-generational HFD exposure on the five basic tastes of offspring. Thus, this study aimed to investigate the combined effect of maternal HFD consumption during pregnancy and lactation, as well as HFD intake in the early life of rat offspring, on the peripheral taste system.

## Materials and Methods

### Animals and experimental design

**Figure 1:**
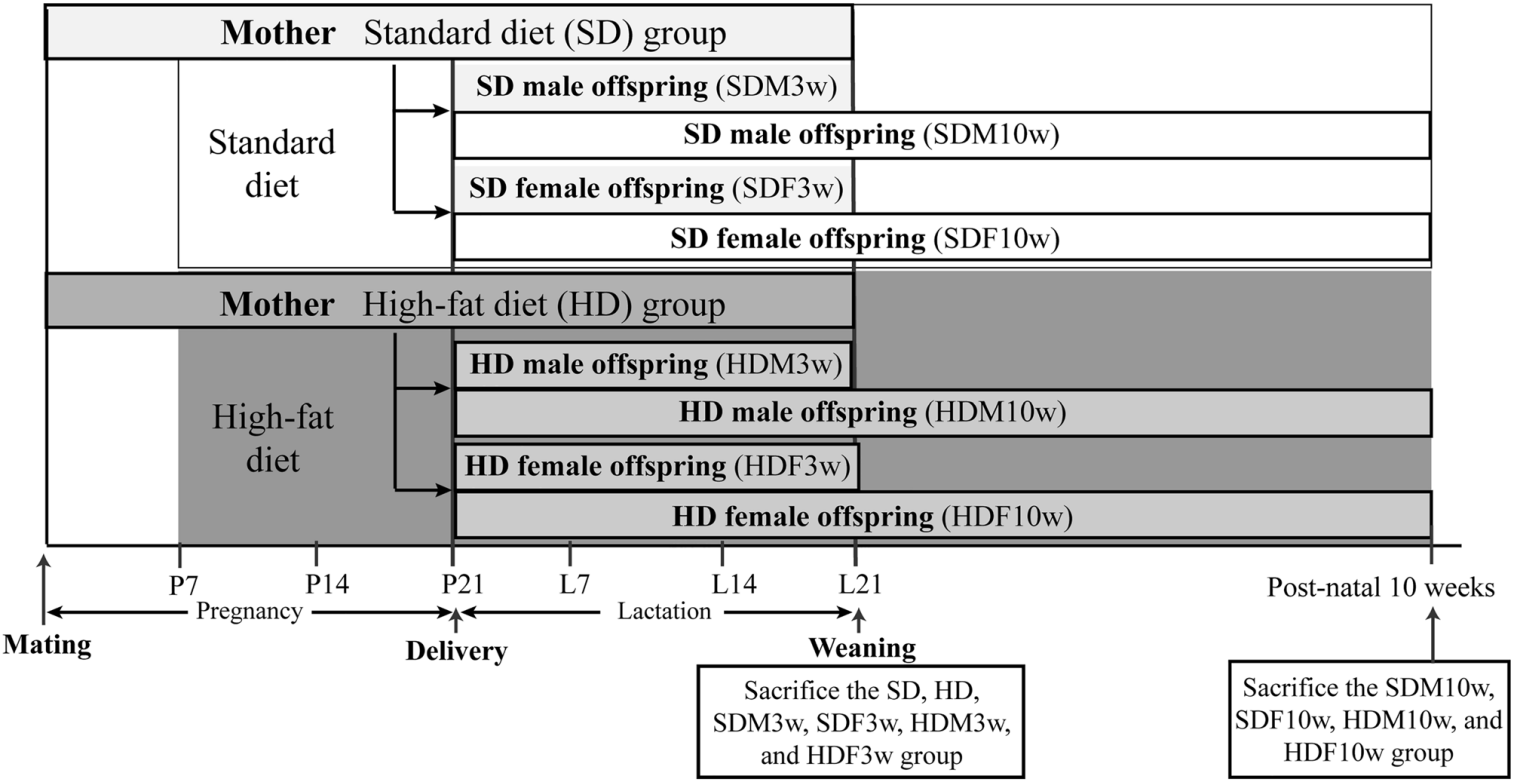
Experimental design. Eleven-week-old pregnant female Wistar rats (n = 10) were divided into the standard diet (SD) and high-fat diet (HFD) groups. The SD group (n = 5) was fed a standard diet and the HFD group (n = 5) was fed a high-fat diet from 7 days of pregnancy (P7) until the end of the lactation period (L21). At delivery, both male and female offspring from the SD and HFD groups were divided into 3-week-old (SDM3w, SDF3w, HDM3w, and HDF3w) and 10-week-old groups (SDM10w, SDF10w, HDM10w, and HDF10w). At weaning, the SD, HFD, SDM3w, SDF3w, HDM3w, and HDF3w were sacrificed. After that, SDM10w and SDF10w were weaned on the same diet as the SD group, and HDM10w and HDF10w were weaned on the same diet as the HFD group. The taste preference behaviors of SDM10w, SDF10w, HDM10w, and HDF10w were analyzed using the 48-h two-bottles preference test. The SDM10w, SDF10w, HDM10w, and HDF10w groups were euthanized at 10 weeks of age.

Eleven-week-old female pregnant Wistar rats (n = 10) were purchased from the Sankyo Labo Service Corporation (Tokyo, Japan). All rats were individually housed in a humidity-controlled room with a 12-h/12-h light/dark cycle and a constant temperature of 23°C. The rats were divided into the SD and HFD groups (Fig. 1). Both SD (n = 5) and HFD groups (n = 5) were fed ad libitum water and either a standard diet (CE2 Clea, Japan; 4.6% from fat, 3.402 kcal/g) or an HFD (HFD32, Clea, Japan; 32% from fat, 5.076 kcal/g), respectively, from 7 days of pregnancy (P7) until the end of the lactation period (L21). After parturition, each mother was caged with their pups until the end of lactation.

Offspring from the SD and HFD groups were randomly divided into two group sets: three-week-old and ten-week-old. The three-week-old group set consisted of nine male and nine female offspring from the SD and HFD groups named SDM3w (n = 9), SDF3w (n = 9), HDM3w (n = 9), and HDF3w (n = 9), respectively. Body weight and fasting blood glucose levels were measured before euthanasia at 3 weeks of age. In addition, the ten-week-old group set consisted of six male six female offspring from the SD and HFD groups assigned to the following groups: SDM10w (n = 6), SDF10w (n = 6), HDM10w (n = 6), and HDF10w (n = 6). The offspring in this group set was weaned on the same diet as their mothers and raised individually per cage in a humidity-controlled room with a 12-h/12-h light/dark cycle and a constant temperature of 23°C. The obesogenic environment was mimicked by the post-weaning HFD. Daily food intake, energy intake, and weekly body weight were measured. The 48-h-two-bottle preference test was performed from three weeks of age until the day of euthanasia at ten weeks of age.

Fasting blood glucose levels were monitored in mothers and offspring. All rats were subjected to daytime fasting (8 h) to minimize stress^26^. Blood samples were collected from the tail vein under inhalation anesthesia (isoflurane; Wako, Japan), and fasting blood glucose levels were measured weekly using a glucometer (Accu-Chek guide; Roche DC, Switzerland).

All animal and experimental procedures were approved by the Institutional Animal Care and Welfare Committee (A2020-148A) prior to the study. In addition, all experimental procedures were conducted according to the Animal Care Standards of Tokyo Medical and Dental University (TMDU) and ARRIVE guidelines.

### Forty-eight-hour two-bottle preference test

During the lactation period, each mother rat in the SD and HFD groups was caged with their pups. Two identical bottles were used for 48 h. After 24 h, the bottle position was switched to prevent positional effects. Initially, rats in the SD and HFD groups were given two bottles of distilled water and trained to drink equally from the bottles. After training for one week, a 48-h two-bottle preference test for sweet taste was conducted from day 14 of the lactation period. The rats were allowed access to two bottles for 48 h; one bottle contained distilled water and the other contained a sweet tastant solution (sucrose; Wako, Japan). The lowest to highest concentrations of sucrose solution (1, 30, and 100 mM) were used for the 48-h-two-bottle preference test^27^. The preference ratio was calculated as follows: tastant solution intake/total solution intake (tastant intake + distilled water intake)^27^.

After weaning, the offspring in the SDM10w, SDF10w, HDM10w, and HDF10w groups were trained for a week. Following that, a two-bottle taste preference test of the five basic tastes was conducted. The concentrations of each tastant solution were given in the following order: bitter: 1, 5, 10 mM denatonium benzoate (denatonium; TCI lot no. CZ2LK-LD, Japan); sour: 1, 5, 10 mM citric acid (Wako, Japan); salty: 30, 100, 300 mM sodium chloride (NaCl) (Wako, Japan); sweet: 1, 30, 100 mM sucrose (Wako, Japan); and umami: 1, 10, 100 mM monosodium glutamate (MSG) (Wako, Japan) with 0.5 mM inosine monophosphate (IMP) (MP Biomedical, Japan). Both SD and HFD groups were subjected to the same procedure. During the one to two days between different tastes, rats were given two bottles of distilled water. The SDM10w, SDF10w, HDM10w, and HDF10w groups were euthanized at ten weeks of age after completing all five tastes of the two-bottle preference test.

### Immunohistochemical staining

Tongue samples from the SDM3w, SDF3w, HDM3w, HDF3w, SDM10w, SDF10w, HDM10w, and HDF10w groups were collected and fixed in 4% formaldehyde (Mildform 10 NM; Wako, Japan) for 24 h. The circumvallate papilla from the tongue was dissected and embedded in paraffin using an automated machine (RH-12DM; Sakura Finetek Japan, Japan). The embedded circumvallate papilla was cut into 5-µm-thick sections.

Sections for angiotensin II receptor type 1 (AT1) immunostaining were deparaffinized in xylene and rehydrated in a descending series of ethanol concentrations. Antigen retrieval was performed using a sodium citrate buffer (pH 6.0). Briefly, the sodium citrate buffer was preheated in a microwave oven, incubated for 20 min, and cooled for an additional 20 min. Thereafter, sections were rinsed in phosphate-buffered saline (PBS) for 2 min and the procedure was repeated. Endogenous peroxidase activity was blocked by incubating with 3% H_2_O_2_ in methanol for 30 min at room temperature. After washing in distilled water for 5 min two times, the sections were blocked in normal serum solution for 30 min and incubated with AT1 (#PA5-2081, Invitrogen, USA; 1:500) primary antibody in 1% bovine serum albumin (BSA) in PBS overnight at 4°C.

To evaluate the expression of amiloride-sensitive epithelial sodium channel alpha (ENaCα) in the circumvallate papilla, deparaffinization, rehydprefen, and antigen retrieval were performed in the same manner as for AT1. The samples were rinsed in Tris-buffered saline containing Tween 20 (TBST) for 3 min three times. The sections were blocked with 3% H_2_O_2_ in methanol for 1 h and washed twice with distilled water for 5 min. The sections were blocked by incubation with 3% BSA in TBST for 2 h and incubated with ENaCα (SPC403D, StressMarq Biosciences, Canada; 1:1000) overnight at 4°C.

Subsequent procedures were performed using the VECTASTAIN^®^ Elite ABC-HRP Kit (PK-6100, Vector Laboratories, USA). The sections were washed in a washing buffer and incubated with biotinylated secondary antibody for 30 min. After rinsing, the sections were incubated with VECTASTAIN^®^ Elite ABC reagent for 30 min and finally washed with a washing buffer. PBS and TBST were used as washing buffers for AT1 and ENaCα immunohistochemistry staining, respectively.

Immunoreactivity was visualized with 3,3′-diaminobenzidine (ab64238, Abcam, USA) for 25 s and rinsed with distilled water for 5 min three times. The sections were counterstained with Mayer’s hematoxylin (Wako, Japan) for 40 s and washed thoroughly with running water for 15 min. Finally, the sections were dehydrated using a series of graded ethanol concentrations, xylene, and thereafter, mounted on coverslips using Mount-Quick (Daido Sangyo, Japan). Sections were visualized using a light microscope (Microphoto-FXA, Nikon, Japan) equipped with a digital camera (DXm1200, Nikon, Japan). The semi-quantitative immunohistochemical analysis of ENaCα and AT1 in the lateral trench wall region was performed by using the imaging software (Image J 1.53f51, NIH, USA) following the protocol described in previous papers^28,29^ (Supplementary Fig. S3). Five images per rat were used to quantify both factors.

### Reverse transcription-quantitative polymerase chain reaction (RT-qPCR)

Total RNA of the circumvallate papilla was extracted using Sepasol-RNA I Super G (Nacalai Tesque, Japan), according to the manufacturer’s protocol. In brief, samples were homogenized in Sepasol-RNA I Super G and kept at the room temperature for 5 min. Chloroform 500 μL was subsequently added and mixed well. After incubating at room temperature for 3 min, tubes were centrifuged at 12,000 × g for 15 min at 4°C. Supernatant was collected and mixed with 500 μL of 2-propanol. After keeping at the room temperature for 10 min, tubes were centrifuged again at 12,000 × g for 10 min at 4°C. Supernatant was discarded and the RNA pallets were washed with 1000 µL ethanol. After centrifugation at 12,000 × g for 5 min at 4°C, supernatant was removed. RNA was quantified by spectrophotometry at 260 nm. RNA was stored frozen at − 80°C until use.

Complementary DNA (cDNA) was synthesized from 1.0 µg of total RNA using ReverTra AceTM qPCR RT Master Mix (Toyobo, Japan), following the manufacturer’s protocol. Real-time quantitative RT-PCR was performed using the StepOne ™ Real-Time PCR System (Applied Biosystems, USA) with TaqMan technology (TaqMan™ Gene Expression Assay, Applied Biosystems) and Probe qPCR Mix (Takara, Japan). The TaqMan probe IDs used were GAPDH (Rn99999916_s1), ENaCα (Rn00580652_m1) and AT1 (Rn01435427_m1). The PCR reaction mixture prepared was 20 µL in total, containing 50 μg of cDNA template, Probe qPCR Mix (1 ×), 0.9 μM of each Taqman Primer, 0.25 μM probe, and ROX reference dye. Amplification of cDNA was performed using the following parameters: 95°C for 20 s and 40 cycles of 95°C for 1 s and 60°C for 20 s. Each assay was carried out in triplicate. The average threshold cycle (Ct) values were analyzed by StepOne™ software (version 2.3; Applied Biosystems, USA) using the 2^−ΔΔCT^ method. GAPDH was used as an internal control to normalize the amount of cDNA loaded. The fold-change in messenger ribonucleic acid (mRNA) expression was relative to that in the control group.

### Statistical analysis

All statistical analyses were performed using SPSS (version 23.0, IBM Corp., Armonk, NY, USA) and GraphPad Prism 9 (GraphPad version 9.4.0, USA). The normal distribution of data was tested using the Shapiro–Wilk test. Statistical significance was set at *p* < 0.05. Data are presented as mean ± standard error of the mean (SEM).

Maternal physiological data were compared between the SD and HFD groups using an unpaired two-tailed Student’s t-test. Two-way repeated-measures analysis of variance (ANOVA) and the Bonferroni test were used to analyze the behavioral assays.

All offspring data were compared based on gender; the SDM10w was compared to the HDM10w, and the SDF10w was compared to the HDF10w using the same statistic as that used for the maternal data. The differences in the offspring’s physiological data were compared using an unpaired two-tailed Student’s t-test and Mann–Whitney U test. The behavioral assays were compared using two-way repeated-measures ANOVA followed by the Bonferroni test.

## Results

### HFD influenced offspring body weight

**Figure 2:**
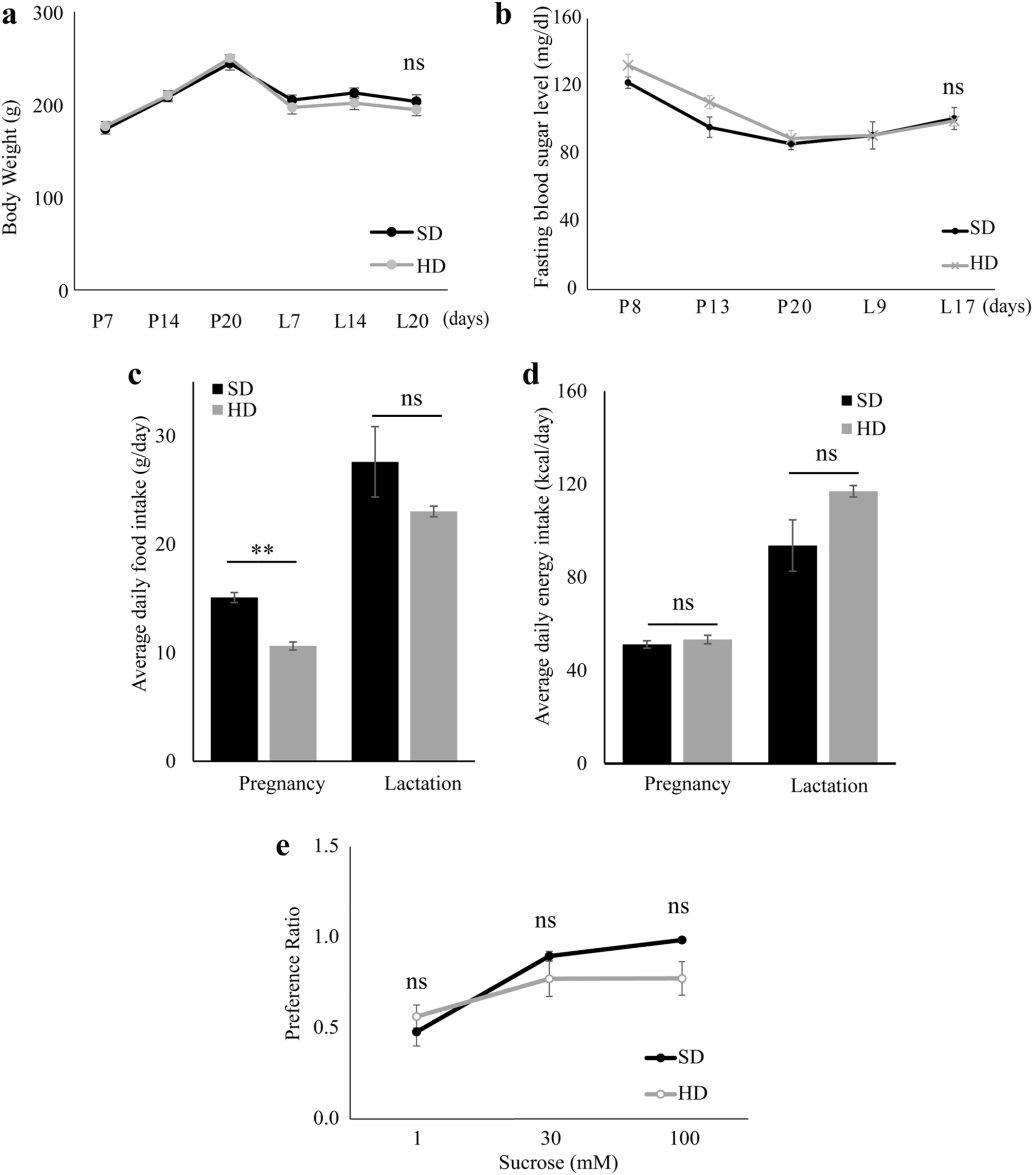
Maternal body weight, fasting blood glucose level, food intake, energy intake, and the preference ratio for sweet taste from the forty-eight-hour two-bottle preference test of SD compared to the HFD group. (**a**) Weekly body weight of the SD and HFD groups throughout the experiment**.** (**b**) Fasting blood glucose level between SD and HFD group**.** (**c**) Average daily food intake of SD and HFD during pregnancy and lactation. (**d**) Average energy intake during pregnancy and lactation period of SD and HFD. (**e**) Preference ratio for the sweet taste of SD and HFD. Data were present as mean ± SEM (**: *p* < 0.001, ns: not significant).

There was no significant difference in body weight between the SD and HFD groups throughout the experiment (Fig. 2a). The body weights of both the SD and HFD groups increased during pregnancy and reached a maximum on day 21 of pregnancy (P21). After delivery, the weight of each group decreased but became stable, and no significant difference between the groups was observed. During 5 weeks of HFD consumption, the fasting blood glucose level (8-h daytime fasting) of the HFD group was not significantly different from that of the SD group during pregnancy and lactation (Fig. 2b). The SD group consumed significantly more food than the HFD group during pregnancy (t (8) = 7.574, *p* < 0.001, n = 5) (Fig. 2c), and the energy intake of the SD and HFD groups was not significantly different during pregnancy and lactation periods (Fig. 2d).

**Figure 3:**
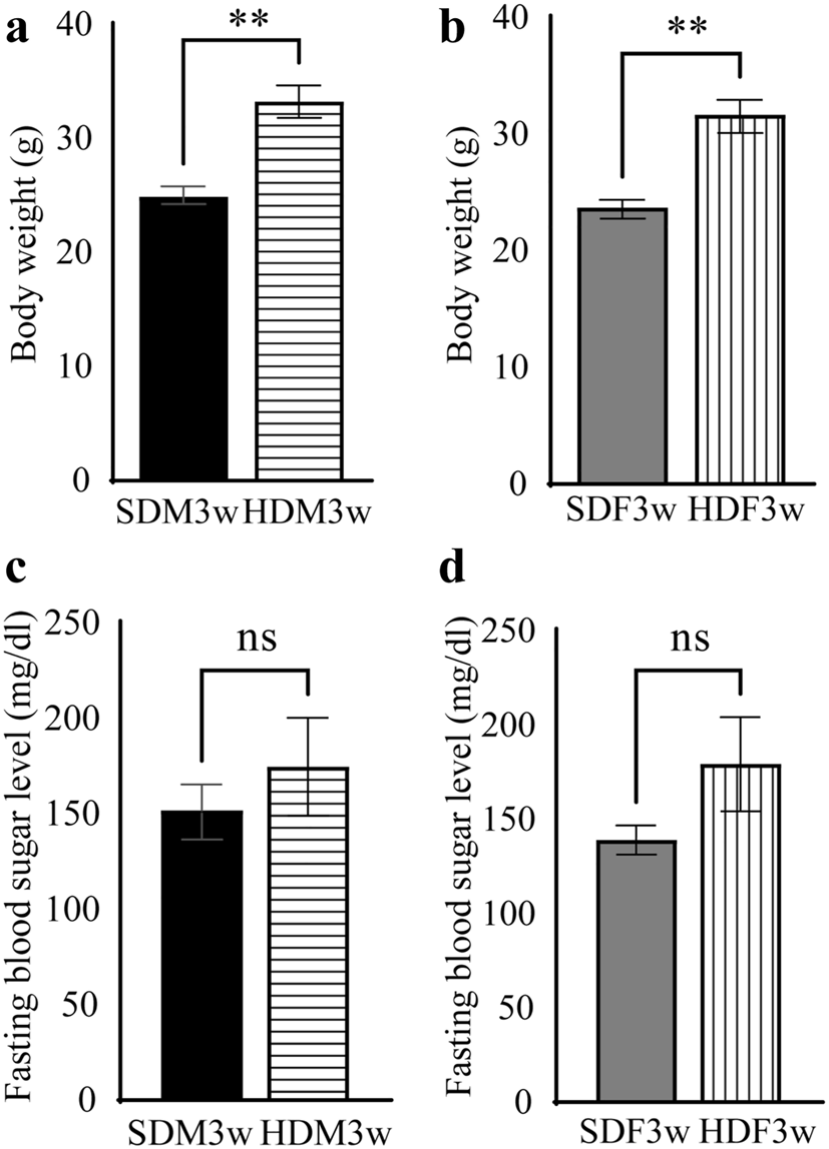
Body weight and fasting blood glucose level of SDM3w, SDF3w, HDM3w, and HDF3w group. (**a**) Body weight of SDM3w compared to HDM3w. (**b**) Body weight of the SDF3w compared to HDF3w. (**c**) Fasting blood glucose level of the SDM3w compared to HDM3w. (**d**) Fasting blood glucose level of SDF3w compared to HDF3w. (**:* p* < 0.001, ns: not significant).

Offspring began receiving milk from parturition until they were three weeks old. As the offspring possibly reached and consumed the mother’s food during late lactation, the SDM3w, SDF3w, HDM3w, and HDF3w groups received the most energy and nutrition from the mother’s milk, while the maternal diet provided only minor nutrition. The weaning body weights of the HDM3w and HDF3w groups were significantly higher than those of the SDM3w and SDF3w groups (U = 1, *p* < 0.01, n = 9) (Fig. 3a, b). However, there was no significant difference in the blood glucose levels between SDM3w, SDF3w, HDM3w, and HDF3w (Fig. 3c and d).

**Figure 4:**
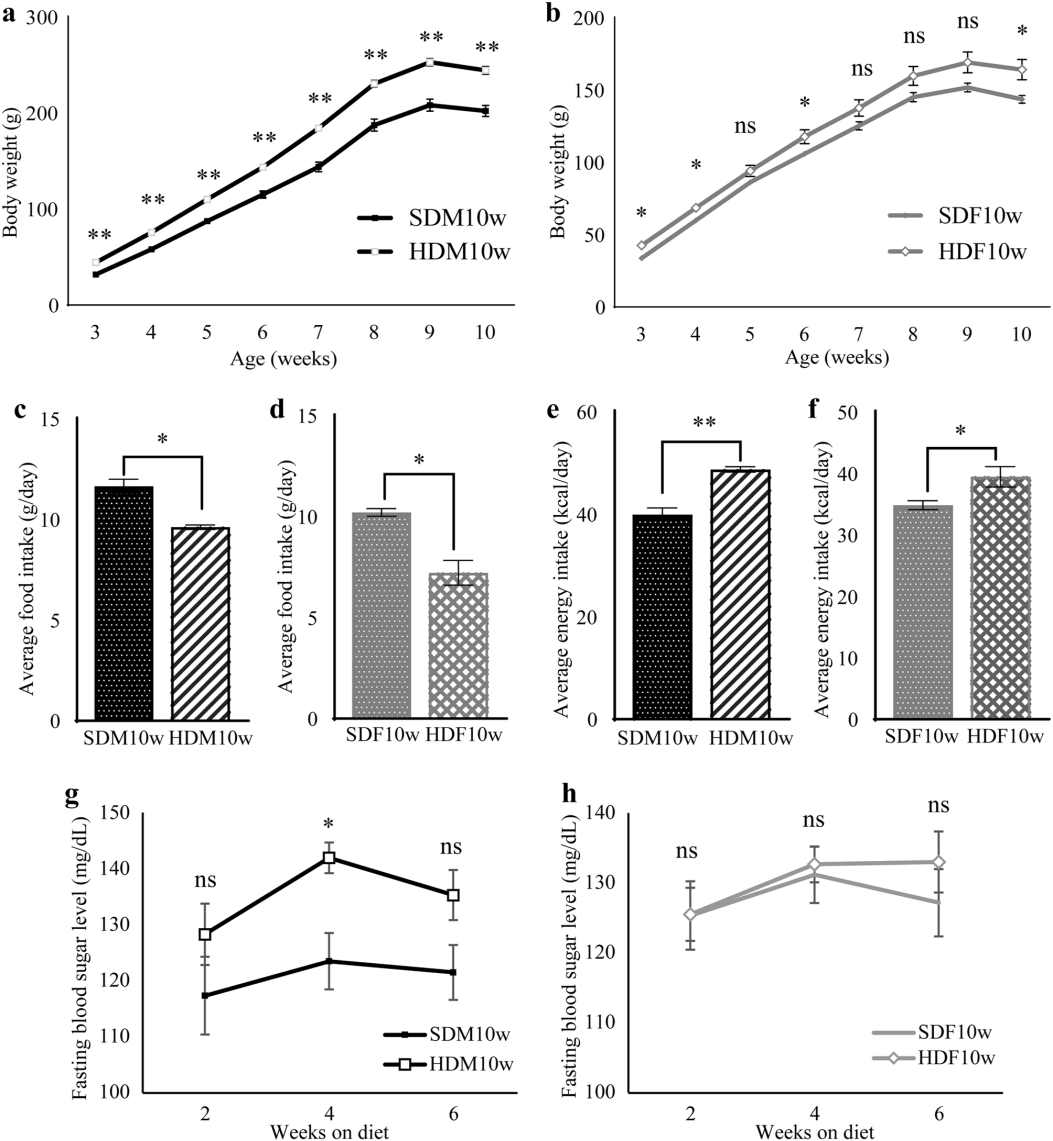
The body weight, food intake, energy intake, and fasting blood glucose level of SDM10w, SDF10w, HDM10w, and HDF10w. (**a**) Weekly body weight of the SDM10w compared to HDM10w. (**b**) Weekly body weight of the SDF10w compared to HDF10w. (**c**) Average food intake of SDM10w compared to HDM10w. (**d**) Average food intake of SDF10w compared to HDF10w. (**e**) The energy intake of SDM10w and HDM10w. (**f**) The energy intake of SDF10w and HDF10w. (**g**) The fasting blood glucose level of SDM10w compared to SDM10w. (**h**) The fasting blood glucose level of SDF10w compared to SDF10w. (*: *p* < 0.05, **: *p* < 0.001, ns: not significant).

In the SDM10w, SDF10w, HDM10w, and HDF10w groups, the weekly body weight was significantly higher in the HDM10W group than in the SDM10w group, throughout the experiment (3 weeks old: t (10) = 6.093, *p* < 0.001; 4 weeks old: t (10) = 7.171, *p* < 0.001; 5 weeks old: t (10) = 7.510, *p* < 0.001; 6 weeks old: t (10) = 6.561, *p* < 0.001; 7 weeks old: t (10) = 7.081, *p* < 0.001; 8 weeks old: t (10) = 6.037, *p* < 0.001; 9 weeks old: t (10) = 7.289, *p* < 0.001; 10 weeks old: t (10) = 7.142, *p* < 0.001, n = 6) (Fig. 4a). Furthermore, the difference increased as the HDM10w and SDM10 grew. In contrast, HDF10w was significantly heavier than SDF10w at 3, 4, 6, and 10 weeks of age (3 weeks old: t (10) = 3.374, *p* = 0.007; 4 weeks old: t (10) = 2.552, *p* = 0.029; 6 weeks old: t (10) = 2.271, *p* = 0.046; 10 weeks old: t (10) = 2.756, *p* = 0.020, n = 6) (Fig. 4b). The HDM10w and HDF10w groups had a less significant consumption of an HFD (HDM10w vs. SDM10w; t (6.032) = 5.717, *p* = 0.001, HDF10w vs. SDF10w; t (10) = 3.221, *p* = 0.001, n = 6) compared to the SDM10w and SDF10w groups, respectively (Fig. 4c, d). However, the energy intake of the HDM10w group was significantly higher than that of the SDM10w group (t (10) = 4.408, *p* < 0.001, n = 6) (Fig. 4e). Similarly, energy intake in the HDF10w group was significantly higher than that of SDF10w (t (10) = 5.156, *p* = 0.028, n = 6) (Fig. 4f). During the six weeks of different diets, fasting blood glucose levels showed no significant difference between the SDF10w and HDF10w groups (Fig. 4h). Nonetheless, the fasting blood glucose level of the HDM10w group was significantly higher than that of the SDM10w in the fourth week of HFD consumption. (t (10) = 3.518, *p* = 0.009, n = 6) (Fig. 4g).

### HFD intake increased salty preference in HDM10w and HDF10w

A 48-h two-bottle preference test was performed to evaluate the alteration in taste preference. There was no significant difference in the preference ratios for sweet taste between the SD and HFD groups during lactation (Fig. 2e).

**Figure 5:**
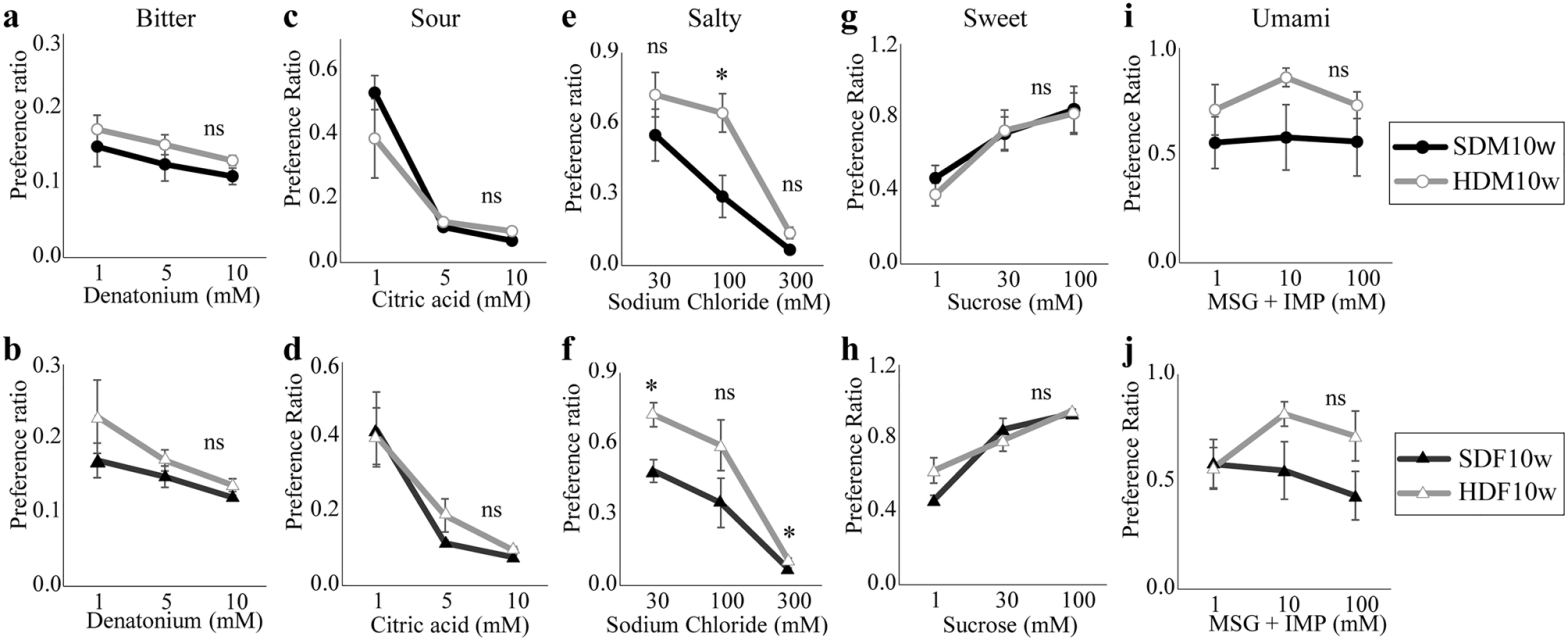
The preference ratio of five basic tastes from the 48-h-two-bottle preference test. (**a**) Preference ratio of the bitter taste of SDM10w compared to HDM10w. (**b**) Preference ratio of the bitter taste of SDF10w compared to HDF10w. (**c**) Preference ratio of the sour taste of SDM10w compared to HDM10w. (**d**) Preference ratio of the sour taste of SDF10w compared to HDF10w. (**e**) Preference ratio of the salty taste of SDM10w compared to HDM10w. (**f**) Preference ratio of the salty taste of SDF10w compared to HDF10w. (**g**) Preference ratio of the sweet taste of SDM10w compared to HDM10w. (**h**) Preference ratio of the sweet taste of SDF10w compared to HDF10w. (**i**) Preference ratio of umami taste of SDM10w compared to HDM10w. (**j**) Preference ratio of umami taste of SDF10w compared to HDF10w. (mean ± SEM, two-way repeated measures ANOVA) (*: *p* < 0.05, ns: not significant).

The preference ratios for salty taste in the HDM10w and HDF10w groups were significantly higher than that in the SDM10w and SDF10w groups, respectively (SDM10w vs HDM10w: F (1, 10) = 6.120, *p* = 0.033; SDF10w vs HDF10w: F (1, 10) = 7.641, *p* = 0.020, n = 6) (Fig. 5e, f). There was no significant difference in the preference ratios for bitter (Fig. 5a, b), sour (Fig. 5c, d), sweet (Fig. 5g, h), and umami (Fig. 5i, j) tastes.

### HFD increased AT1 expression in HDF3w in immunohistochemical staining

Based on the alteration in salty taste preference behavior of the HDM10w and HDF10w groups, we further evaluated factors associated with the perception of salty taste, namely, ENaC and AT1. ENaC, the membrane ion channel responsible for salty taste, has an alpha subunit that is mostly abundant in the circumvallate papilla^30^. AT1 is one of the main angiotensin II receptors and has been reported to be associated with upregulated salty taste preference.^31^.

**Figure 6:**
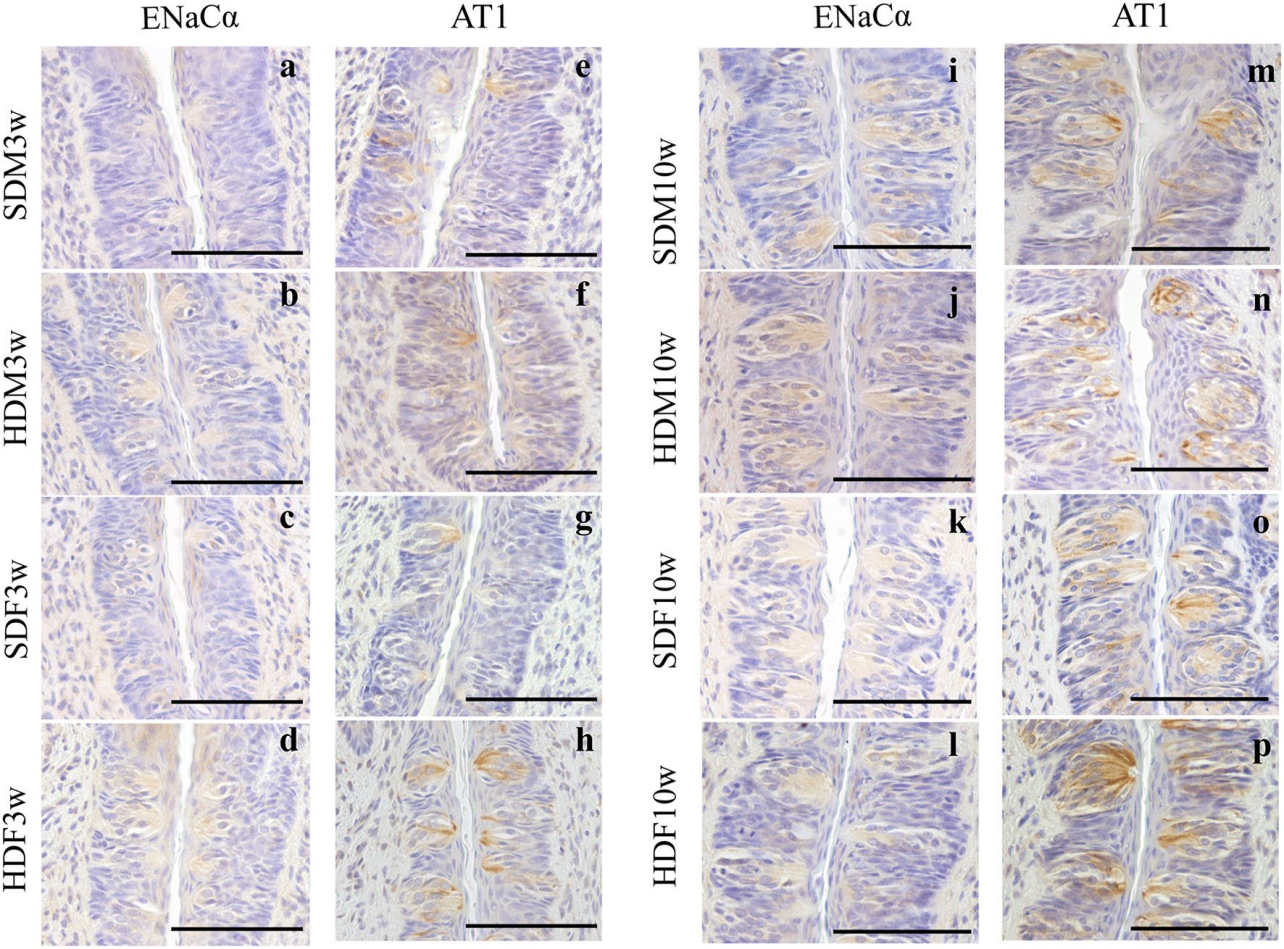
Immunohistochemical staining of ENaCα and AT1 in the circumvallate papilla of offspring. (**a–d**) Immunohistochemical staining of ENaCα in the circumvallate papilla of SDM3w, HDM3w, SDF3w, and HDF3w. (**e–h**) Immunohistochemical staining of AT1 in the circumvallate papilla of SDM3w, HDM3w, SDF3w, and HDF3w. (**i–l**) Immunohistochemical staining of ENaCα in the circumvallate papilla of SDM10w, HDM10w, SDF10w, and HDF10w. (**m–p**) Immunohistochemical staining of AT1 in the circumvallate papilla of SDM10w, HDM10w, SDF10w, and HDF10w. Scale bars = 100 μm.

Immunohistochemical staining revealed a high density of ENaCα immunoreactivity on the epithelial surface of the circumvallate papilla and lower density inside the taste bud cells of the SDM3w, SDF3w, HDM3w, and HDF3w groups (Fig. 6a–d and Supplementary Fig. S1). In contrast, AT1 was expressed in circumvallate taste buds but not in the epithelium. AT1 was predominantly expressed at the apical side of taste bud cells, which were close to the taste pore and in contact with the oral cavity environment (Fig. 6e–h and Supplementary Fig. S1). The area of AT1 immunoreactivity in the HDF3w group was more prominent than that in the SDF3w group.

**Figure 7:**
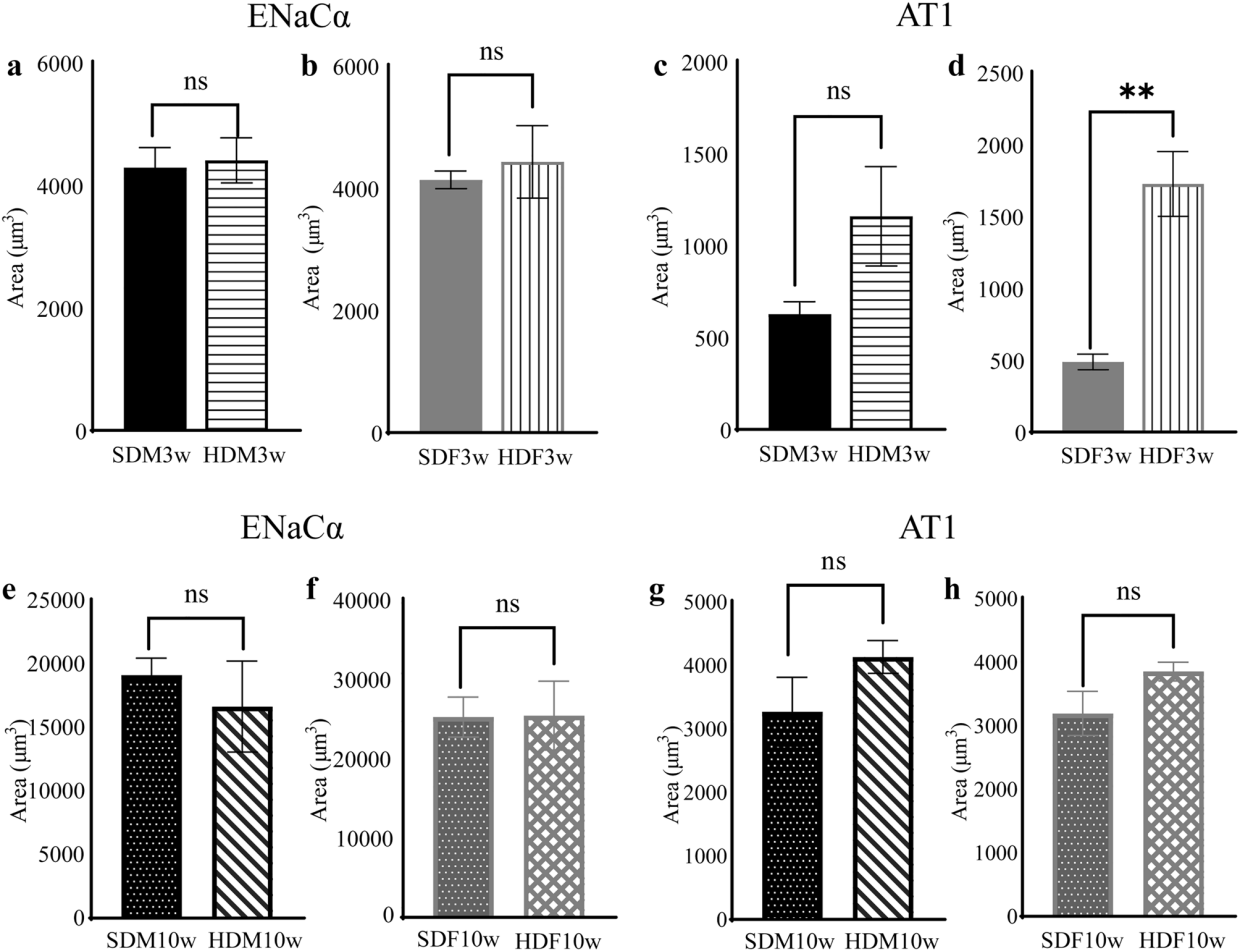
Semi-quantitative immunohistochemical analysis of ENaCα and AT1 in the lateral trenches of circumvallate papilla of offspring. (**a**) The ENaCα area of SDM3w compared to HDM3w. (**b**) The ENaCα area of SDF3w compared to HDF3w. (**c**) The AT1 area of SDM3w compared to HDM3w. (**d**) The AT1 area of SDF3w compared to HDF3w. (**e**) The ENaCα area of SDM10w compared to HDM10w. (**f**) The ENaCα area of SDF10w compared to HDF10w. (**g**) The AT1 area of SDM10w compared to HDM10w. (**h**) The AT1 area of SDF10w compared to HDF10w.

ENaCα expression was observed in the epithelium and taste bud cells of circumvallate papillae of the SDM10w, SDF10w, HDM10w, and HDF10w groups (Fig. 6i–l and Supplementary Fig. S2). ENaCα expressions in the taste buds of SDM10w, SDF10w, HDM10w, and HDF10w groups were more prominent than those in the taste buds of the SDM3w, SDF3w, HDM3w, and HDF3w groups. In addition, AT1 was detected only in the taste bud cells of the SDM10w, SDF10w, HDM10w, and HDF10w groups and was more remarkable than that in the SDM3w, SDF3w, HDM3w, and HDF3w groups (Fig. 6m–p and Supplementary Fig. S2). The semi-quantification of ENaCα and AT1 showed an increase in AT positive staining area in HDF3w compared to SDF3w (U = 0, *p* < 0.001, n = 3) (Fig. 7d). There was no significant difference among other groups (Fig. 7).

### HFD increased the level of AT1 gene expression of HDF3w

**Figure 8:**
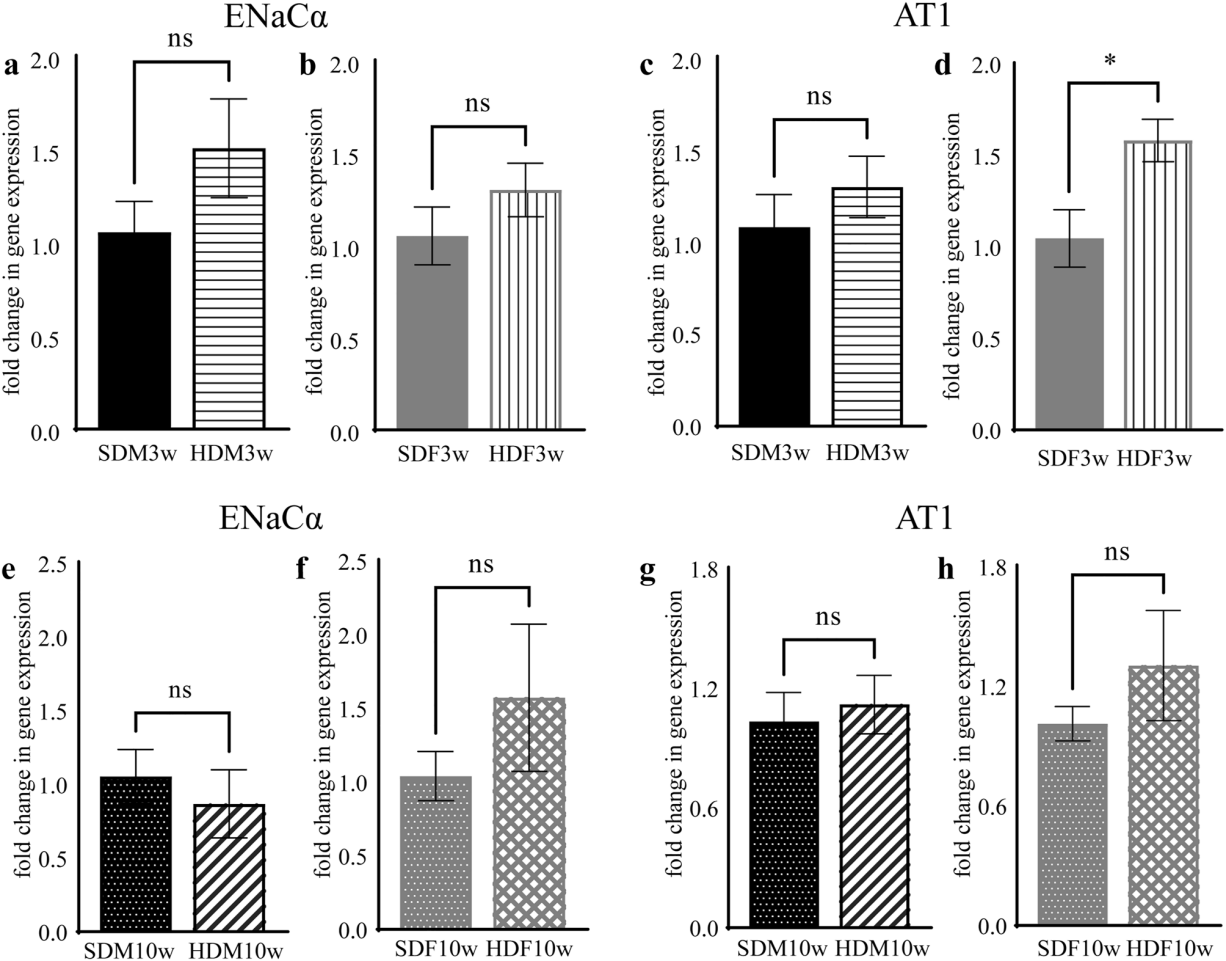
The mRNA expression of ENaCα and AT1 in the circumvallate papilla of offspring. (**a**) The level of ENaCα between SDM3w and HDM3w. (**b**) The level of ENaCα between SDF3w and HDF3w. (**c**) The level of AT1 of SDM3w compared to HDM3w. (**d**) The level of AT1 of SDF3w compared to HDF3w. (**e**) The level of ENaCα between SDM10w and HDM10w. (**f**) The level of ENaCα between SDF10w and HDF10w. (**g**) The level of AT1 of SDM10w compared to HDM10w. (**h**) The level of AT1 of SDF10w compared to HDF10w. (*: *p* < 0.05, ns: not significant).

The mRNA expression levels of ENaCα and AT1 were determined by RT-qPCR. The fold change in gene expression of AT1 confirmed the immunohistochemical staining results. There was a significantly higher expression of AT1 mRNA in the HDF3w group (1.585-fold) than in the SDF3w group (t (10) = 2.751, *p* = 0.0204, n = 6) (Fig. 8d). Nevertheless, AT1 expressions in the HDM3w and SDM3w groups were not significantly different (Fig. 8c). ENaCα mRNA levels in HDM3w and HDF3w groups were not significantly different compared to SDM3w and SDF3w, respectively (Fig. 8a, b). Furthermore, the expression of ENaCα and AT1 in the SDM10w, SDF10w, HDM10w, and HDF10w groups was not significantly different (Fig. 8e–h).

## Discussion

In this study, we observed the taste preference alteration in the two-generational HFD exposure group by behavioral test and analyzed the involved mechanisms. Previous research has shown that maternal HFD alters the taste preference and sensitivity in offspring^23,29^. Moreover, a post-weaning HFD exacerbated the hyperphagia character in offspring^24,32^. We hypothesized that maternal and post-weaning HFD exposure aggravate the adverse effect in offspring. However, most previous studies mainly focused on maternal HFD during pregnancy and lactation on control offspring^23,29,32^. In most cases, the food pattern of individuals in postnatal life is similar to the parental food pattern^33,34^. Therefore, we used the same methods as those of previous researchers^15,35^ and examined the effect of two-generation exposure to an HFD on offspring taste. The salty taste alterations were observed in both the HDM10w and HDF10w groups. Although maternal sweet taste preference during pregnancy was not significantly different between the SD and HFD groups (Fig. 2e), we discovered a higher salty taste preference in the HDM10w and HDF10w groups than in the SDM10w and SDF10w groups (Fig. 5e, f). This phenomenon was particularly important when we further investigated the expression of ENaCα and AT1. An upregulated AT1 in the HDF3w group (Fig. 8d) implied the underlying factor leading to the higher salt preference in the HDF10w group.

HFD-fed rats, specifically the HFD, HDM10w, and HDF10w groups, consumed significantly less food than the control group. This phenomenon could be caused by post-ingestion negative feedback from high-fat foods, a signal from the digestive tract that reduces appetite to control meal size and compensate for the higher energy of HFD food^36–38^. Because the energy value in an HFD was higher than that in standard food (standard food: CE2 Clea, Japan; 3.4 kcal/g vs. HFD: HFD32, Clea, Japan; 5.1 kcal/g), the HFD and SD groups eventually received similar energy values during pregnancy and lactation and maintained the same body weight. In the second generation, the HDM10w and HDF10w groups consumed less food than the SDM10w and SDF10w groups; however, this was insufficient to compensate for the higher caloric value of the HFD and received significantly more energy during the growth stage (Fig. 4c–f). Higher energy intake resulted in correlated body weight results. The increase in energy intake in the HDM10w group over the SDM10w group was greater than the increase in the HDF10w group over the SDF10w group (Fig. 4e, f). Consequently, the higher body weight in the HDM10w group compared to that in the SDM10w group was consistently observed, while the higher body weight in the HDF10w group compared to that in the SDF10w group was inconsistently observed throughout the experiment (Fig. 4a, b). Post-ingestive negative feedback in offspring was insufficient to compensate for the high calories of an HFD that could result from the long duration of HFD consumption. Chronic HFD exposure may impair leptin hormone response and result in excessive food intake^38^.

HFD intake had no effect on fasting blood glucose levels in the HFD group when compared to the SD group (Fig. 2b) or in the HDF10w group when compared to the SDF10w group (Fig. 4h). However, after 4 weeks of consumption, there was an increase in the blood glucose levels in the HDM10w group compared to that in the SDM10w group (Fig. 4g). A previous study suggested that a high intake of saturated fat, but not unsaturated fat, could cause insulin resistance and diabetes^39,40^. However, the HFD used in this experiment contained 66.5% monounsaturated fat, 10.4% polyunsaturated fat, and 22.3% saturated fat. Therefore, the higher percentage of unsaturated fat could explain the indifferent blood glucose levels or insulin resistance in the SD and HFD groups.

The lactation period is critical for newborn mammals^41^. An imbalance in nutrition in maternal milk may result in developmental programming effects, affecting the health of the offspring throughout life^42^. After weaning, the body weight of the offspring, which mainly received energy and nutrition from the mother’s milk, was significantly higher in the HDM3w and HDF3w groups than in the SDM3w and SDF3w groups (Fig. 3a, b). Our results demonstrate that milk from HFD-fed mothers influence and significantly increases the body weight of their offspring. Previous animal studies have found that an HFD before pregnancy and during lactation causes changes in milk content^41^. Milk from HFD-fed mother rats had lower water content and higher fat content^41^. The increased fat from the milk of the HFD group could explain why pups in the HDM3w and HDF3w groups were significantly heavier than those in the SDM3w and SDF3w groups.

To the best of our knowledge, no studies have screened for preference alterations in the five basic tastes in the offspring after two generations of exposure to an HFD. After screening the five basic taste preferences, we discovered that HFD altered the salty, but not the bitter, sour, sweet, and umami taste preference behavior of the HDM10w and HDF10w groups. The HDM10w and HDF10w groups had an increased selection for salty taste compared with the SDM10w and SDF10w groups, respectively. Although salt does not provide energy, it is significantly associated with obesity in humans^43–45^. Previous studies have reported reduced salty taste sensitivity in obese individuals compared to lean individuals, with obese individuals requiring higher salt intake to sense the salty taste^46,47^. Higher sugar-sweetened beverage consumption, induced by salt intake, is potentially linked to obesity^48^. Moreover, previous studies have reported that higher salt intake is directly associated with higher body fat mass in children and adults^43,44^. In line with previous studies, both HDM10w and HDF10w groups had a higher salty taste preference along with a higher body weight. A previous study suggested that increased activity in specific brain regions, which is related to taste sensation, food memory, and appetite, may be one of the mechanisms contributing to the alteration of salty taste preference in obese patients^47^. However, dissimilar results were observed, probably due to methodological differences among studies. Some studies reported no significant difference in salty taste preference between non-obese and obese patients^49,50^, whereas other studies found the opposite, with lower salt sensitivity in obese patients^51,52^.

Various organs are involved in detecting stimuli at taste receptors, sending signals via nerves, and interpreting them in the brain^53^. Taste signals begin at the taste buds distributed on the tongue. Circumvallate papillae are located on the posterior part in the midline of the tongue and surrounded by the circumferential groove. Cross-sections of circumvallate papillae display taste buds localized in the lateral trench wall region^54^. Taste buds are garlic-shaped, connected to the oral cavity via the small opening called “taste pore,” and contain 50–100 taste bud cells inside (Supplementary Fig. S3). Every taste bud can detect all five basic tastes similarly regardless of the position^20,55^. In salty taste perception, sodium (Na^+^) is detected by taste cells via ENaC^20^. ENaC expressed in taste bud cells and the adjacent epithelium are considered to be involved in salty taste sensation^20,56^. ENaC in rodents consists of three subunits, alpha (ENaCα), beta (ENaCβ), and gamma (ENaCγ), which have been found to differ in expression in the different areas of the tongue^30,57^. ENaCα is mostly expressed in the posterior tongue^30^. Furthermore, a series of studies have indicated that angiotensin II induces an increased preference for salt, especially sodium ions^57–59^. Some authors have also reported that renin-angiotensin system components, renin, angiotensinogen, and angiotensin-converting enzyme-1, including AT1, are present in fungiform papillae and circumvallate papillae^31^. This evidence suggests that taste buds may produce angiotensin II and influence taste bud cells via AT1 receptors^31,60^. As a result, we chose to study the expression of ENaCα and AT1 in the circumvallate papilla of the offspring, since it is the most prominent and contains the highest number of taste buds^22,61^. Immunohistochemical staining demonstrated ENaCα and AT1 expression in the circumvallate papillae with different characteristics. The immunopositive area for AT1 was mostly present in the taste bud cells, especially near the taste pore, and became denser in the SDM10w, SDF10w, HDM10w, and HDF10w groups than in the SDM3w, SDF3w, HDM3w, and HDF3w groups, respectively. On the other hand, ENaCα was expressed in the taste bud cells and epithelium without taste bud cells and was expressed more strongly in the taste buds of SDM10w, SDF10w, HDM10w, and HDF10w than in the SDM3w, SDF3w, HDM3w, and HDF3w groups, respectively. These results, combined with data from previous studies^31,56,57^, support the idea that ENaCα and AT1 are responsible for the salty taste perception at the circumvallate papillae.

We further investigated the sodium receptors ENaCα and AT1 in the circumvallate papilla using RT-qPCR in the SDM3w, SDF3w, HDM3w, HDF3w, SDM10w, SDF10w, HDM10w, and HDF10w groups. We demonstrated that the expression level of ENaCα was similar in all groups, although the salty taste preference behavior was higher in the HDM10w and HDF10w groups. This suggests that an HFD exposure does not change the amount of ENaCα in the circumvallate papilla after weaning, but possibly alters other factors in the taste system. In contrast, the level of AT1 in the HDF3w group was higher than that in the SDF3w group, while there was no significant difference between the HDM3w and SDM3w groups. The higher expression of AT1 in the HDF3w group at the age of 3 weeks could be a peripheral factor contributing to the increased salty taste preference in the HDF10w group. Angiotensin II increases sodium intake via AT1 in taste cells.^60^ Correspondingly, an increased AT1 expression in the HDF3w, but not in the HDM3w, is consistent with the behavioral test results. A higher salt preference was exhibited at concentrations of 10 mM and 300 mM sodium chloride in the HDF10w group. In comparison, a higher salt preference was observed only at a specific 30 mM sodium chloride concentration in the HDM10w group. Sex differences in salty taste in rats have been reported for decades^62,63^. Adult female rats typically preferred sodium chloride over male rats^63–65^. This sex difference is involved with steroid sex hormones^64^. Previous studies reported that gonadectomy eliminated the sex difference in salt intake^63,64^. Particularly, estrogen can suppress the nerve responses to sodium chloride and consequently decrease salty taste sensitivity^64^. Therefore, the higher salty taste preference in females from our results could be enhanced by the nature of female hormones. Furthermore, an increased AT1 level in the circumvallate papillae in HDF3w suggested that the higher salty taste preference could be associated with the renin-angiotensin system, rather than ENaCα in the posterior of the tongue.

The level of AT1 did not change in the SDM10w, SDF10w, HDM10w, and HDF10w groups at 10 weeks of age. Due to the nature of the taste bud cells, which are continuously renewed every 10–14 days throughout life^66^, it is assumed that the AT1 level in the taste cells adapted during taste bud cells replacement. Notably, circumvallate papillae at the posterior tongue and other types of taste buds in the anterior tongue exhibit different characteristics. The posterior tongue has mostly amiloride-insensitive ENaCs and responds widely to Na^+^, K^+^, and H^+^. In contrast, the anterior part of the tongue mainly contains amiloride-sensitive ENaCs and responds specifically to Na^+^ perception. Moreover, different subunits of amiloride-sensitive ENaCs are localized in the anterior and posterior parts of the tongue. ENaCα, ENaCβ, and ENaCγ are mainly expressed in the anterior tongue, whereas only the ENaCα is easily found in the posterior tongue^57^. Therefore, the results of this study suggest the necessity of further studies on the expression of other ENaC subunits in different types of taste bud cells to understand the underlying mechanisms of taste preference behavioral changes.

Early-stage taste programming has been reported in several studies in both humans and animals^24,25,32,42,67^. In humans, the flavors that the mother is exposed to during pregnancy or lactation could increase the positive response or recognition of the infant^42^. In animal studies, unbalanced maternal nutrition during pregnancy and lactation can lead to overeating behavior and altered taste perception in the offspring^25,32^^,^^23–25,67,68^. From our results, an increase in AT1 in young female offspring is most likely to enhance sodium intake. This is especially important if it occurs in childhood because various types of food are introduced in preadolescence when children start to choose the food by themselves. Furthermore, a higher salty preference in young adults could lead to an excessive sodium intake. Notably, high sodium consumption can increase the risk of systemic complications, including stomach cancer, metabolic syndrome, autoimmunity, kidney stones, osteoporosis, cardiovascular disease, and obesity^69^. High sodium intake also promotes adipocyte size, leptin resistance, and impaired metabolism, leading to obesity^70^. In addition, high salt consumption directly impairs cardiovascular health^71^. Changes in vascular structure and function are the most likely mechanisms. High salt intake directly increases the TGF-β1, a key factor in the promotion of hypertension and vascular and glomerular fibrosis^71^. Understanding the changes in eating habits in offspring exposed to an HFD during fetal and postnatal life could lead to further studies to reduce the risk of obesity and cardiovascular diseases in the direct offspring and the next generation. In conclusion, early taste learning was critical in programming offspring’s eating behaviors and food selection. Therefore, it is crucial to comprehend the complications that arise in offspring’s taste perception at an early stage.

Our findings should be interpreted in light of some limitations. Firstly, the objective of the post-weaning HFD was to mimic an obesogenic environment in offspring. Therefore, the results in this study were not a single effect either from maternal HFD or offspring’s HFD consumption. Secondly, the taste system changes throughout life in rodents^72^. Therefore, age may influence taste preference behavior. Finally, the differences in lactation between rodents and humans are critical. Humans are generally pregnant with one fetus, whereas rodents are pregnant with large litters. As a result, the nutritional requirements for rodents to raise their young are significantly higher than those for humans. These limitations should be considered when predicting their effects in humans.

## Conclusions

HFD exposure over two generations increased AT1 expression in female offspring at weaning and behaviorally increased salty taste preference in male and female rat offspring. Increased salty taste preference can lead to excessive sodium intake and exacerbate sodium homeostasis. Furthermore, alterations in the taste system are most likely to influence eating behavior. Unhealthy eating behavior in offspring increases the risk of lifelong systemic disorders.

## Supplementary Information

Supplementary Figures.

## Data Availability

The datasets generated during and/or analyzed during the current study are available from the corresponding author on reasonable request.

## Abbreviations

AT1: Angiotensin II receptor type 1
ENaCα: Amiloride-sensitive epithelial sodium channel alpha
P: Pregnancy
L: Lactation
MSG: Monosodium glutamate
IMP: Inosine monophosphate
