# Supplementary material for: Two-generation exposure to a high-fat diet induces the change of salty taste preference in rats
Source: Sci Rep. 2023 Apr 7;13:5742. doi: 10.1038/s41598-023-31662-0 (PMC10082214; doi:10.1038/s41598-023-31662-0)
Supplement: Supplementary file 1 — Supplementary Figures. [file 41598_2023_31662_MOESM1_ESM.pdf]

TWO-GENERATION EXPOSURE TO A HIGH-FAT DIET INDUCES THE CHANGE OF SALTY TASTE PREFERENCE IN RATS

Saranya Serirukchutarungsee[1,2], Ippei Watari[1]*, Masataka Narukawa[3], Katarzyna Anna Podyma-Inoue[5], Pornchanok Sangsuriyothai[1,4], Takashi Ono[1]

[1]Department of Orthodontic Science, Graduate School of Medical and Dental Sciences, Tokyo Medical and Dental University (TMDU), Tokyo, Japan

[2]Department of Pedodontics and Preventive Dentistry, Faculty of Dentistry, Srinakharinwirot University, Bangkok, Thailand

[3]Department of Food and Nutrition, Kyoto Women's University, Kyoto, Japan

[4]Department of Orthodontics, Faculty of Dentistry, Chulalongkorn University, Bangkok, Thailand

[5]Department of Biochemistry, Graduate School of Medical and Dental Sciences, Tokyo Medical and Dental University (TMDU), Tokyo, Japan

***** Corresponding author at: Department of Orthodontic Science, Tokyo Medical and Dental University, 113-8510, Yushima 1-5-45, Bunkyo City, Tokyo, Japan
Email address: ippeiwatari@gmail.com

Supplementary Materials

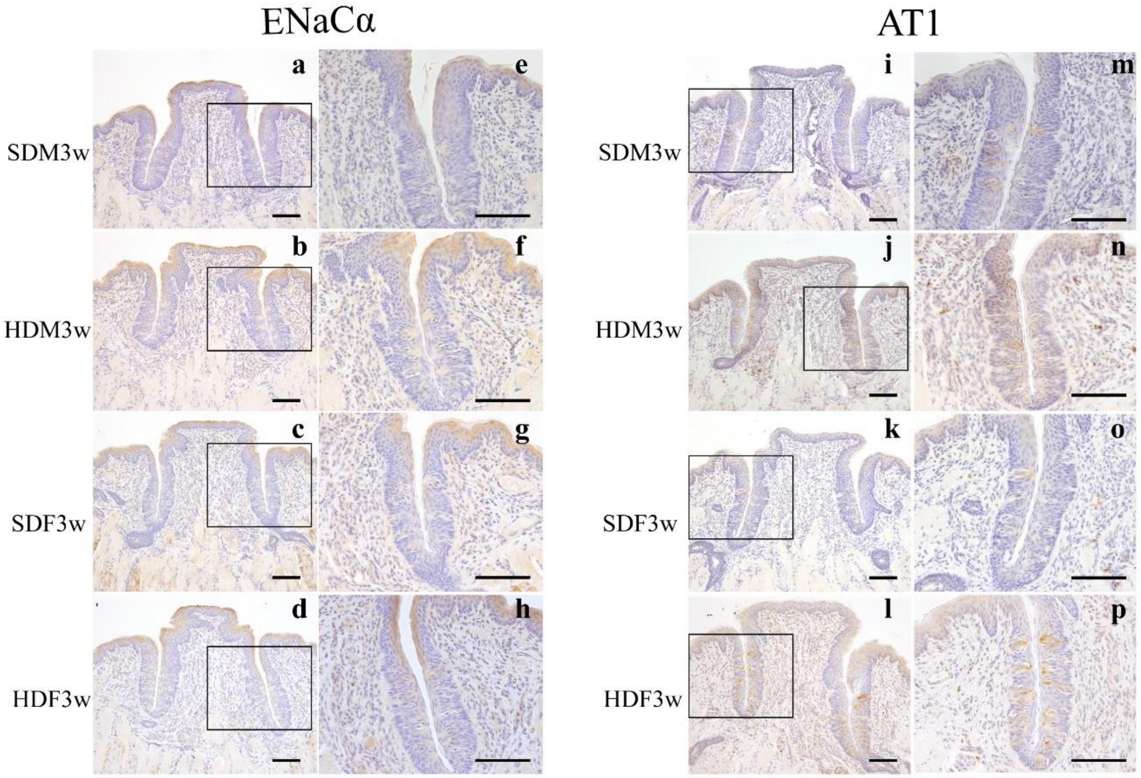

Supplementary figure 1. Immunohistochemical staining of ENaCα and AT1 in the circumvallate papilla of the 3-week-old offspring. **(a-d)** Immunohistochemical staining of ENaCα in SDM3w, HDM3w, SDF3w, and HDF3w. **(e-h)** are the magnification of **(a-d)**. **(i-l)** Immunohistochemical staining of AT1 in SDM3w, HDM3w, SDF3w, and HDF3w. **(m-p)** are the magnification of **(i-l)**. Scale bars = 100 μm

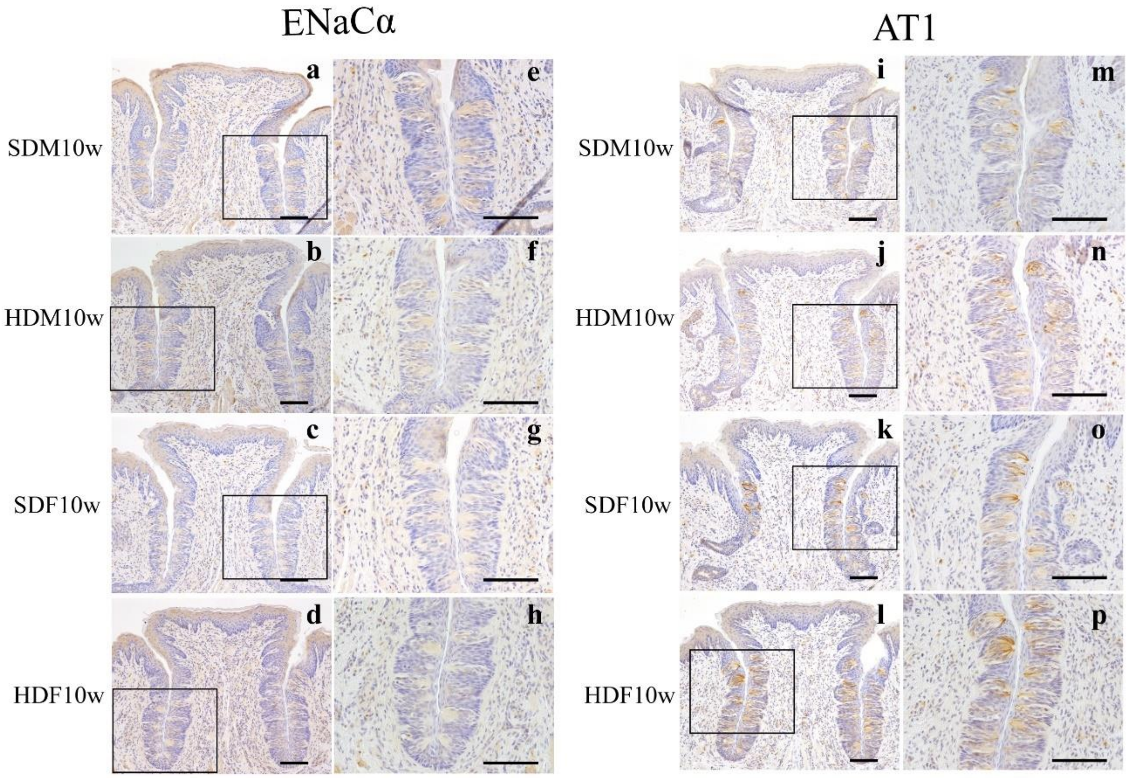

Supplementary figure 2. Immunohistochemical staining of ENaCα and AT1 in the circumvallate papilla of the 10-week-old offspring. **(a-d)** Immunohistochemical staining of ENaCα in SDM10w, HDM10w, SDF10w, and HDF10w. **(e-h)** are the magnification of **(a-d)**. **(i-l)** Immunohistochemical staining of AT1 in SDM10w, HDM10w, SDF10w, and HDF10w. **(m-p)** are the magnification of **(i-l)**. Scale bars = 100 μm

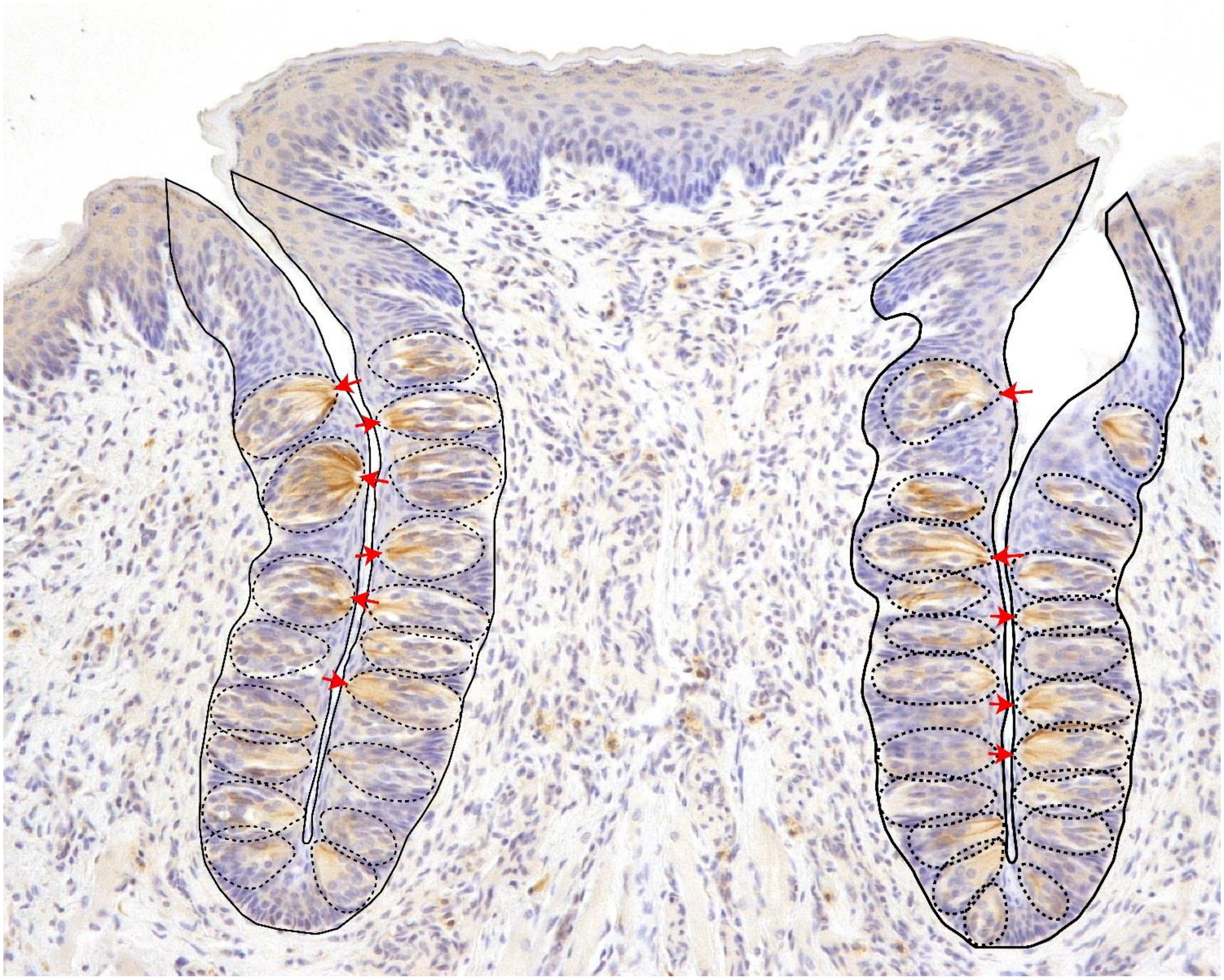

Supplementary figure 3. The trench area and taste bud cells area in circumvallate papillae. The trench area was the epithelium area of the inner to outer trench walls. Black solid line demonstrated the left and right trench areas. The garlic-shaped taste buds embedded in the trench area (dashed line) and connected to the oral cavity via the small opening called "taste pores" (red arrow).
